# Leveraging big data to uncover the eco-evolutionary factors shaping behavioural development

**DOI:** 10.1098/rspb.2022.2115

**Published:** 2023-02-08

**Authors:** Sean M. Ehlman, Ulrike Scherer, David Bierbach, Fritz A. Francisco, Kate L. Laskowski, Jens Krause, Max Wolf

**Affiliations:** ^1^ SCIoI Excellence Cluster, 10587 Berlin, Germany; ^2^ Faculty of Life Sciences, Humboldt University, 10117 Berlin, Germany; ^3^ Department of Fish Biology, Fisheries, and Aquaculture, Leibniz Institute of Freshwater Ecology and Inland Fisheries, 12587 Berlin, Germany; ^4^ Department of Evolution and Ecology, University of California – Davis, Davis, CA 95616, USA

**Keywords:** behavioural development, big data, individuality, personality, tracking

## Abstract

Mapping the eco-evolutionary factors shaping the development of animals’ behavioural phenotypes remains a great challenge. Recent advances in ‘big behavioural data’ research—the high-resolution tracking of individuals and the harnessing of that data with powerful analytical tools—have vastly improved our ability to measure and model developing behavioural phenotypes. Applied to the study of behavioural ontogeny, the unfolding of whole behavioural repertoires can be mapped in unprecedented detail with relative ease. This overcomes long-standing experimental bottlenecks and heralds a surge of studies that more finely define and explore behavioural–experiential trajectories across development. In this review, we first provide a brief guide to state-of-the-art approaches that allow the collection and analysis of high-resolution behavioural data across development. We then outline how such approaches can be used to address key issues regarding the ecological and evolutionary factors shaping behavioural development: developmental feedbacks between behaviour and underlying states, early life effects and behavioural transitions, and information integration across development.

## 1. Background

During the last decade, the rapid emergence of ‘big behavioural data’ research—the high-resolution tracking of individuals and the harnessing of that data with powerful machine learning techniques [1–6]—has spurred major advances in fields as diverse as behavioural genomics and transcriptomics [7–9], behavioural neuroscience [10–15], collective decision-making [16–19] and movement ecology [20–23]. Huge untapped potential remains, however, in applying big data approaches to understand the development of behaviour. In sharp contrast to previous data-limited approaches where only subsets of animals’ developmental trajectories are measured, big behavioural data research allows near-continuous monitoring of animals throughout their entire (or substantial parts of) development. We believe that this innovation, paired with timely advancements in computation, data storage, and tailored analytical tools, represents a watershed moment for understanding the ecology and evolution of behavioural development.

In our review, we begin (§2) by briefly reviewing the state-of-the-art approaches that allow the collection and analysis of behavioural data at increasing resolution. Then, we discuss in detail (§3) how these innovations in measuring and modelling behaviour can be productively applied to fundamental open questions regarding the rules governing the unfolding of behaviour: mapping the nature of developmental feedbacks between behaviour and underlying states, understanding early-life effects and behavioural transitions across development, and understanding information integration across development. Big behavioural data have greatly expanded the empirical toolkit with which to investigate these fundamental issues in behavioural development, and they have also spurred a new generation of conceptual models of behaviour. Throughout, we focus on the links between contemporary and potential future empirical advances and the set of theory and technical models that this new wealth of data can inform and test. Indeed, many of the state-of-the-art methods in behavioural analysis that we review are increasingly simple to use and ready to deploy, but their application to existing theory in behavioural development is largely unrealized; quick progress in this area is thus achievable, as we will outline. In sum, we believe that this mutual feedback between new data sources and novel theory will generate major new insights into one of the foundational elements of the study of behaviour, behavioural ontogeny.

## 2. New methods, measures and means of defining behaviour

One inherent difficulty with measuring the ontogeny of most traits—behaviour among them—is that, for many organisms, these traits often take considerable time to fully develop. Historically, behavioural researchers have tried to circumvent this issue in several ways (table 1): by limiting the period in development over which a trait is measured; by measuring a trait during relatively short periods during development interspersed by larger intervals of time without measurements; by manipulating early-life conditions and investigating the consequences later in life; and/or by adopting comparative approaches across individuals of different ages. These methods, while providing targeted information about certain periods during development or coarse-grained information about the arc of development, may miss critical time windows and complex nonlinearities in behavioural development, thereby hampering our ability to gain a fuller understanding of the factors shaping behavioural development. Additionally, constraints on data collection have also meant that most studies focus on the development of one or a few behavioural traits and thus miss out on quantifying biologically meaningful correlations in developmental time series of suites of traits.

**Table 1.** A stylized overview of the main methods used to study the development of behaviour, including near-continuous behavioural measurements throughout development, that advances in tracking, computational and analytical tools have recently made possible.

methods of quantifying behavioural development	example references
(1) concentrated measurements early in life 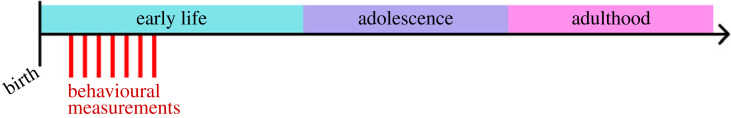	[24,25]
(2) periodic measurements throughout development 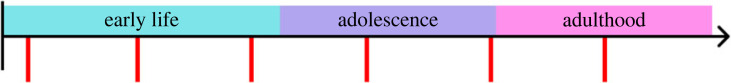	[26–30]
(3) experimental manipulation early in life, behavioural measurements before/after 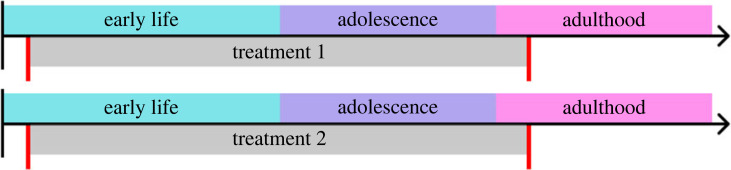	[31–35]
(4) comparative approach across individuals of different ages 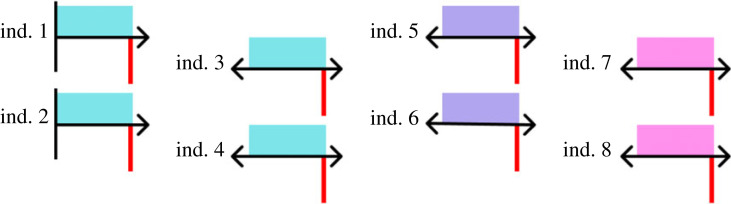	[29,36–38]
(5) near-continuous behavioural measurements throughout development 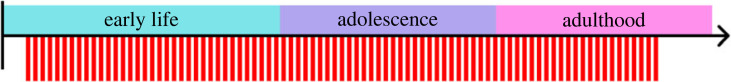	[39,40]

Whereas collecting highly time-resolved data on behaviour through the entire course of development was once often prohibited by daunting logistics and limits on time and effort, the advent of sophisticated automated tracking technology, more advanced data storage infrastructure, and a host of powerful, data-hungry analytical tools can produce and parse these datasets with relatively low cost in terms of both person-hours and monetary investment [10,41,42]. We are now at a critical juncture where early studies have proven the worth of these new technologies for quantifying and conceptualizing behaviour in new ways [10,43–46], but where heretofore surprisingly limited consideration has been paid to the application of these tools to behavioural development. For the remainder of this section, we focus on recent innovations in both the measuring and modelling of behaviour that lay the foundation for the application of these technologies to answer major open questions in behavioural development.

### (a) Measurement innovations

Behavioural tracking technology has come a long way in the last decade, with sub-second temporal resolution in movement data now being standard for many automated real-time trackers. Such datasets, often as simple as a time series of two- or three-dimensional coordinate points in space, are commonly obtained via GPS devices, PIT tags, or video tracking, and numerous tracking software automate the process of converting coordinate point data into behavioural metrics such as activity rates, space use, velocity, etc. [2,6,47–50]. Crucially, many of these tools are specifically designed for keeping track of individuals through time, even when observed in groups, allowing behavioural patterns of many individuals to be tracked simultaneously [6,48,49]. Furthermore, rapid advances are being made in the identification and tracking of postural movements of body segments, allowing studies of fine motor development of social interactions, mating displays, resource manipulation, etc. [2,51]. Such approaches have vastly increased both the temporal resolution and overall duration of data that can be collected, allowing animals to be tracked throughout development with minimal intervention. Tracking technology is not just limited to tracking animal behaviour during development: some of the greatest promise lies in pairing high-resolution behavioural tracking data with complementary, non-behavioural data types with similar resolution. For instance, high-resolution behavioural data coupled with other high-throughput data sources have provided insight into both the neurological [14,52] and transcriptomic correlates of behaviour [8]. Internal state variables such as body condition can be estimated directly from video frames in the case of video tracking [53]. High-resolution physiological data can be obtained from a quickly expanding array of non-invasive biologging tools [54,55], such as heart rate monitors [56,57] or thermal imaging [58–60], and may help infer stress levels, respiration rate or energetic state when coupled with behavioural data. Other approaches use both supervised and unsupervised machine learning methods to infer underlying internal state variables from high-resolution behavioural tracking data themselves [61–64]. We believe there is also tremendous value in the simultaneous tracking of salient environmental variables and behaviour [65–67], allowing researchers to map detailed behavioural–experiential trajectories (i.e. interactions between behaviours and the sequence of experiences that result from interacting with the environment) and track environmental drivers of behaviour throughout development, getting us a step closer to understanding the ecological forces shaping behavioural development.

### (b) Data-driven definitions of behavioural axes

Behavioural biologists have long wrestled with how to identify and classify behavioural ‘traits’ [68,69], and while novel methods of measuring and conceptualizing behaviour may not be a panacea, they offer progress. Equipped with highly resolved behavioural time series over ontogeny, researchers have a quickly growing toolset of analytical techniques that permit, for example, high-throughput auto-labelling of observer-defined behaviours [70], unsupervised learning of new behavioural classes, detection of hierarchical sub-structure in behaviour, or modelling of behavioural transition rates across a wide range of timescales [10,12,42,71].

Big behavioural datasets are large and often inherently multi-dimensional, and while researchers may opt for dramatically reducing the dimensionality of these datasets by, for instance, calculating simple behavioural metrics directly from raw data (e.g. activity rates, velocity, position in relation to focal point or social partner, etc.), a range of tools can now be applied that retain the high dimensionality of these datasets to the degree that it is informative, thus moving a step closer to quantifying the development of integrated behavioural repertoires. While the specific toolkit in parsing big behavioural datasets will vary depending on the dataset and the questions to be answered, a common challenge among most analyses is reducing collinearities in the data structure while maintaining informative variation and interpretability. This task—determining the salient axes over which behaviours vary through ontogeny—sets the stage for then delimiting specific sets of related behaviours (also known as behavioural ‘classes’ or ‘clusters’) along these axes.

One of the simplest approaches for extracting behavioural axes in large, highly dimensional datasets is principal component analysis (PCA) [72]. More sophisticated techniques such as spectral analysis [73] or time frequency analysis [43,45] are specifically designed for time-series analysis and have also been productively applied to behavioural time series as a step towards defining the axes of behavioural variation across development. The important point is that, rather than researchers pre-defining which behavioural axes are important, the primary goal of these techniques is to leverage variation in the data themselves in order to determine axes of behavioural variation among a suite of (often correlated) behavioural metrics. In a dataset of bee behaviours across their adult lifetime, for instance, PCA was applied to a set of metrics derived from tracking data (e.g. location in the hive and movement metrics such as speed and space use), allowing the authors to avoid redundancies among many potentially non-independent metrics and instead define orthogonal behavioural axes ordered by the degree to which they explained variation in the overall dataset [39]. PC axes and more complex, nonlinear dimension reduction techniques [74,75] are also highly useful as visualization tools: high-dimensional behaviour such as specific posture patterns [43,44,46,76,77], social interactions [78] or microhabitat-related behaviours [79] have all been mapped using computational approaches to defining behavioural axes. The diversity of tools in this toolset allows researchers to accommodate the many unique challenges that might arise when, for example, behavioural covariance structures change across development (e.g. thus affecting the ‘loadings’ in a PCA), or when trade-offs between mapping ‘local’ versus ‘global’ behavioural variation are rebalanced [80].

Using statistical techniques to define the major axes of variation in behavioural time-series data has the potential to offer insights into fundamental questions about the structure and development of individual behavioural variation, that is, individuality. Traditionally, studies on behavioural individuality and animal personality have sought to describe behavioural variation along researcher-defined axes such as ‘boldness’, ‘exploration’ and ‘sociality’, a modification of the ‘Big Five’ model of personality variation imported from human psychology [81]. While this tradition is now firmly embedded in animal personality research, a steady flow of critique has questioned both the wisdom of defining traits *a priori* (e.g. whether something like ‘boldness’ or ‘exploration’ holds the same meaning across diverse species) and the methodological *status quo* of measuring behaviours along pre-defined axes (e.g. whether a certain assay actually measures ‘boldness’, ‘exploration’, etc.) [69,82–84]. Data-driven statistical approaches to defining behavioural axes have indeed been offered as alternatives to pre-defining such axes when quantifying individuality [85,86] (but see [87]), and the growing availability of high-dimensional behavioural datasets increases their appeal. Such methods may allow more comparability across periods of development, populations or even species, by first inferring the behavioural axes using data from all developmental time points/populations/species in an analysis and then asking how time points/populations/species differ across these ‘latent’ axes.

### (c) Identifying behavioural classes and quantifying transitions among classes

Once the axes of behavioural variation are identified, an important next step is delimiting how different behaviours distributed along these axes interrelate to each other, creating a ‘map’ of the structure of observed behavioural variation. Again, there are a diversity of methods to achieve this; however, one exciting set of methods, unsupervised behavioural classifiers (i.e. clustering algorithms), are increasingly being applied to big behavioural datasets as tools to define behavioural classes—groups of behavioural variables with high similarity [63,80,88,89]. Clustering, while not necessarily appropriate for all big behavioural datasets or questions, excels as a tool for dividing large, multi-dimensional behavioural data trait-spaces into interpretable subregions based on similarity, distance, or regions of high density in data space. In many cases, researchers may choose to forgo clustering: behavioural plasticity, for example, could be quantified without defining clusters and instead measuring ‘movement’ or ‘behavioural entropy’ in a multi-dimensional ‘behavioural space’. On the other hand, delineating clusters—or discrete groups of behaviours—may become useful when, for example, researchers are interested in understanding transition rates among distinct regions of behavioural trait-space throughout development. Used as tools to define behavioural diversity in entire behavioural repertoires, such methods are essentially a way to generate rich, data-driven ethograms.

A host of clustering algorithms has been deployed in unsupervised classification of behaviour, but many take as an input a specified and arbitrary number of clusters (i.e. behavioural classes), *k*, in a multi-dimensional data space [80]. While one might treat the decision about the magnitude of *k* as an optimization problem, many of the behavioural trait-spaces that are created with high-dimensional, dense behavioural data may not show clear, discrete boundaries between identified clusters [39]; in such cases, *k* is perhaps most useful as a parameter that is actively toggled to adjust the ‘graininess’ of behavioural partitioning. Indeed, creative, flexible use of *k* as a free parameter enables researchers to examine the structure or ‘architecture’ of behavioural repertoires. For example, Berman *et al.* [44] showed that, as they increased the number of clusters, *k*, new clusters were created largely by subdividing existing clusters in two (rather than more substantially reshuffling the behavioural map); this led the researchers to conclude that observed fly behaviours were structured hierarchically, testing decades-old theory in ethology [90,91].

Finally, once distinct behavioural clusters/classes are defined, the transitions among these classes can be quantified. One of the simplest and most common approaches to modelling behavioural transitions is to assume that behavioural transitions follow a Markovian process. These models assume that the probabilities of transitioning to any other behavioural state (i.e. cluster) depend only on an animal's current behaviour, rather than some sequence of behaviours that preceded the current behaviour. Common extensions of this concept are hidden Markov models (HMMs) whereby a sequence of behavioural patterns (e.g. movement) are linked to an underlying unmeasured (behavioural) state variable (e.g. a specific behavioural class such as foraging, hiding, walking and running). In this way, HMMs can be important tools in identifying behavioural modes [73,92], similar to behavioural clusters discussed earlier. Recently, HMMs have been extended to include nested latent states in order to explore hierarchical structures in behavioural modes (hierarchical hidden Markov models, HHMMs) [93,94]—an approach that is likely to be useful in high-resolution behavioural development data that can be parsed at a large range of temporal scales.

**Figure 1.**
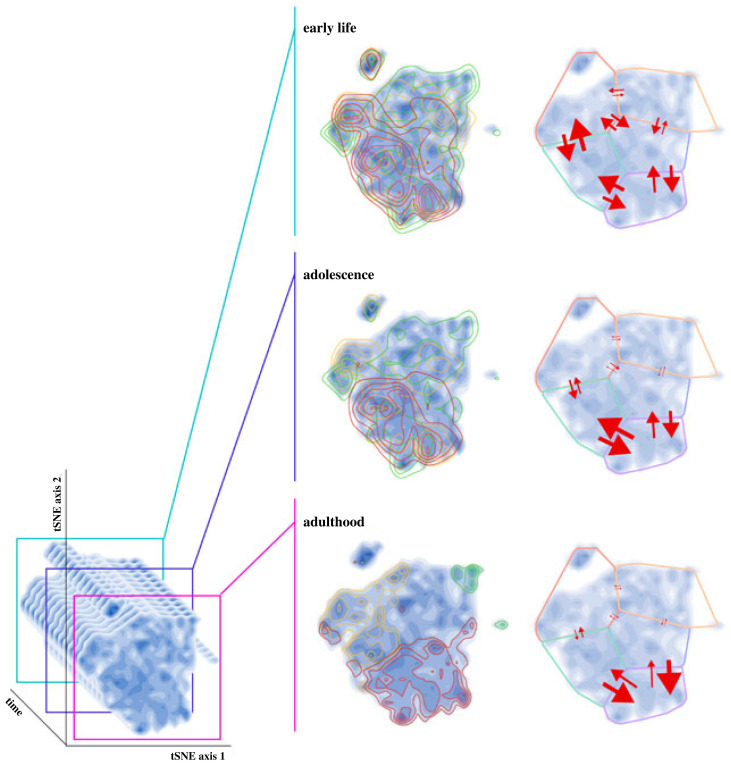
After dominant behavioural axes have been calculated (e.g. via PCA), multi-dimensional behavioural data for all individuals and all developmental time points can be projected onto a two-dimensional behavioural/phenotypic space (e.g. via tSNE (*t*-distributed stochastic neighbour embedding), UMAP (uniform manifold approximation and projection) or other dimension reduction tools). Individual behavioural repertoires during subsets of development can then be characterized (here with contour plots of three individuals in green, yellow and red shown only for early life, adolescence and adulthood for ease of visualization; note that high-temporal resolution allows much finer-grained analyses across many more individuals), and further quantification of individual behavioural transition rates among distinct behavioural classes/clusters can be conducted (represented for the ‘red’ individual with arrows for which size and thickness are proportional to the magnitude of transition probabilities between different behavioural clusters). In this example, individuals explore a large range of behavioural space early in development but become more constrained—and distinct from each other—as development progresses. Such a pattern of decreasing behavioural plasticity across development is a major prediction of many Bayesian models of development, and a pattern of behavioural divergence among individuals across development is consistent with behavioural niche specialization [95,96]. See [43,44] for worked examples of many of the above approaches in a non-developmental context.

By determining the axes of behavioural variation, mapping onto these axes distinct behavioural classes or clusters, and then quantifying transitions among these classes, we can begin to ask questions that shed light on behavioural changes throughout development (figure 1): how do transitions between behaviours (i.e. clusters) change during development? Are there certain regions of this ‘behavioural map’ that are more frequently used by newborns versus adolescents versus adults? Are there certain areas of behavioural space that are only accessible to animals after a certain developmental time point? Do individuals decrease or increase in the amount of behavioural ‘space’ they use throughout their lifetime? Are individuals consistently different in how they ‘move through behavioural space’ during development? While many of these questions have been asked (and addressed) in some form previously, the degree of precision and depth with which these questions can now be addressed—not just for particular behaviours but for much of an animal's entire movement repertoire—is unprecedented.

## 3. Using big behavioural data to uncover the principles governing behavioural development

We will now discuss how big behavioural data and the associated innovations in measuring and modelling behaviour discussed above can be productively applied to fundamental open questions regarding the principles governing behavioural development.

### (a) Mapping behaviour–state connections across ontogeny

Connections between behavioural decisions and an animal's underlying state (e.g. energetic state, informational state, social rank, etc.) may play a major role in the development of behaviour: such connections can help explain the evolution and development of a large range of behaviours and states (e.g. foraging tactics [97,98], anti-predator behaviours [99–101], emotions and self-awareness [102,103], developmental adjustments in response to environmental change [104,105]). While theoretical and conceptual models employing techniques such as stochastic dynamic programming [106] have hugely facilitated the investigation of behaviour–state connections and their role in the development of adaptive behaviour [107–112], empirical studies that simultaneously measure behaviour and state throughout development are relatively few. As discussed above (see §2*a*, ‘Measurement innovations’), behavioural tracking techniques provide a powerful way to directly measure state variables (or proxies of state variables) with as much automation as behaviour, allowing direct tests of the wealth of theory on behaviour–state connections with high-resolution behaviour and state time series across development.

**Figure 2.**
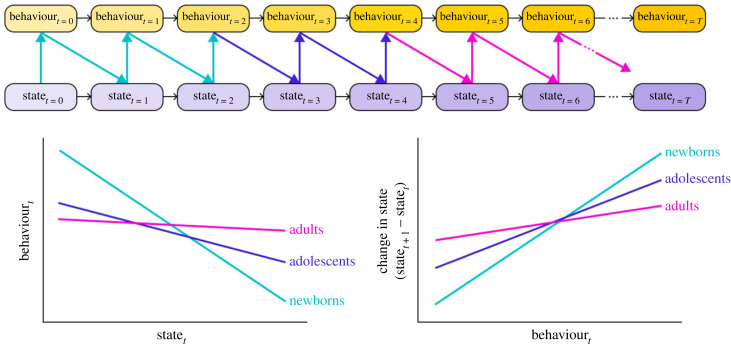
High-resolution behavioural tracking paired with similarly high-resolution tracking of state variables allow tests that detect behaviour–state feedbacks and quantify how both the strength and direction of these feedbacks change across development. In this schematic, for example, negative feedbacks between behaviour and state persist throughout development, but are stronger earlier in life (blue–green lines represent individuals in early development, purple in mid-development and magenta at maturity; note that with highly temporally resolved datasets, such an analysis could be done more continuously).

One major open question is how particular types of behaviour–state connections such as dynamic feedback loops (i.e. bidirectional linkages between state and behaviour) influence developmental trajectories [113–115]. So far, of the studies that have been designed to detect behaviour–state feedbacks, conclusions on the presence and consequences of these feedbacks have been mixed [116–119]. Nevertheless, behaviour–state feedbacks are commonly cited in the literature as a major causal factor in the formation of behavioural developmental trajectories. The now possible simultaneous high-resolution tracking of both behaviour and state can allow us to (i) detect the presence of feedbacks and (ii) measure the persistence of both the strength and direction of these feedbacks across development at a much finer temporal grain. To be concrete, consider a high-resolution time series of behaviours and states, where at each point in time *t*, *B_t_* and *S_t_* represent behavioural and state measurements, respectively (figure 2). Behaviour–state feedbacks would be evident if *S_t_* correlated with *B_t_ and* *B_t_* was then correlated with a change in state (i.e. Δ*S_t_* = *S_t_*_+ 1_ – *S_t_*) at some future point in time, *t* + 1. As an example, one could imagine that energetic state (e.g. fat reserves or hunger level) would affect foraging behaviour at one time point and that the intensity of any foraging behaviour would then subsequently be related to a further change in energetic state at the following time point. Furthermore, by testing for these correlations over subsets of a developmental time series, one could test for the degree to which feedbacks persist in both strength and direction throughout development. Such an investigation could test whether the slopes in the relationship between *S_t_* and *B_t_* and *B_t_* and Δ*S_t_* vary as *t* increases, and whether any changes are symmetrically represented in both relationships.

One commonly cited behaviour–state feedback mechanism is the asset-protection principle [120]: if having high ‘state’ (here, residual reproductive value) makes it less likely that an individual will take risks (here, actions that are associated with increased mortality but potentially increased state benefits, e.g. foraging under predation risk), a negative feedback might be generated [121,122]. Low-state individuals would thus take more risks compared with high-state individuals, thus increasing their state and becoming less likely to take further risks. The strength of this negative feedback may be expected to change over development (figure 2). For example, prey outgrowing a dominant gape-limited predator or gradually learning how to better evade predators may represent particular ecologies that generate systematic decreases in risk—and hence the strength of the negative feedback—through a prey's lifetime [123]. Figure 2 illustrates such a scenario where temporally high-resolution behaviour and state tracking allow us to test for the change in behaviour–state feedback strength across development: while negative feedbacks, indicated by slopes of the relationships between *S_t_* and *B_t_* and *B_t_* and Δ*S_t_* having opposing signs, persist throughout development, the absolute values of the slopes of these lines—and thus the strength of feedbacks—decrease through life. Thus, the ability to detect behaviour–state feedbacks and quantify how the strengths of these feedbacks change through time will afford us greater potential to map the rules governing the unfolding of developmental trajectories to the specific selective forces particular to a species’ ecology.

### (b) Early-life effects and behavioural transitions

Continuous tracking of behaviour during development is particularly promising for understanding early-life effects [124,125]. Such early-life effects (e.g. parental imprinting or social experience during early life stages [126–128], the influence of early-life stressors on later behaviour [129,130], etc.) are typically studied experimentally by manipulating environmental stimuli early in life and then measuring outcomes (e.g. behaviour) later in life (table 1, example 3). The focus on early life has been particularly intense relative to other periods of animal life because (i) the developmental consequences of phenotypic adjustments made early in development may be outsized compared with those of later adjustments (e.g. the epiphenotype hypothesis [131]), (ii) animals may show greater developmental plasticity early in life [107], perhaps reflecting adaptive strategies in environments where animals gradually accrue fitness-related information from steady streams of imperfect cues [132], and (iii) early-life experiences often have long-lasting consequences for behaviour and fitness [133–136], with effects even persisting across multiple generations [137]. Despite their importance, however, the developmental routes connecting early-life experiences and their long-term outcomes often remain unmeasured and thus obscured.

Experiments based on near-continuous tracking throughout development will allow us to unravel the behavioural mechanisms that link early-life experiences to outcomes later in life. High-resolution tracking will provide both longer and more highly resolved time series of behavioural data that measure the entire developmental time course of responses to salient early-life stressors or stimuli. Whereas previous approaches might capture gross patterns or periods of sensitivity to cues in a few key periods of development, high-throughput behavioural measurements could more closely estimate the exact ‘cue–response curve’ [138]. Of key interest will be the periods of life near ‘switching points’ in cue sensitivity when animals switch from being sensitive to cue input to being unresponsive to cues. A highly temporally resolved dataset could then address, for example, whether these switching points represent a rapid switch, which may signal a threshold-mediated response or criticality in the underlying cue–response systems [139], or a more graded change in cue sensitivity, as one might observe during many learning processes [111,140]. Furthermore, it is around these switching points that individual variation is likely to exist; differences in the timing and magnitude of cue responses to ecological factors will further our understanding of the ecological and evolutionary factors driving individual variation.

While switching points often represent relatively brief and intense periods of behavioural transition, individuals are also transitioning among behavioural states on more frequent timescales second-by-second and day-by-day. Such state-switching is another important facet of behavioural development and—as we have discussed above (see §2*c*, ‘Identifying behavioural classes and quantifying transitions among classes’)—can now be captured by high-resolution behavioural tracking. And while state-switching models that calculate transition probabilities among behavioural states have been applied to behavioural data for decades, relatively large temporal graininess has only allowed transitions between high-level behavioural categories to be modelled [141,142]. By contrast, the richness of datasets produced through most automated tracking techniques allows much more sub-structure in behaviour to be modelled. For example, whereas a relatively low-resolution time series may allow the estimation of transition rates on the scale of a day (i.e. estimating the probability of behavioural shifts from one day to the next throughout development), the highly resolved datasets typical of automated tracking technology not only enable the estimation of those same day-to-day transition rates with greater accuracy, but also allow the estimation of transition rates between behaviours that occur on finer scales of hours or minutes [143–147].

Leveraging previously discussed approaches to define and classify behaviour (see §2*b*, ‘Data-driven definitions of behavioural axes’, and §2*c*, ‘Identifying behavioural classes and quantifying transitions among classes’), transition models (including Markov models) will allow us to address fundamental questions concerning the structure of behavioural development: when and to what extent are behavioural patterns during development path-dependent? Are behaviours operating at different timescales structured hierarchically (i.e. do distinct behavioural patterns consist of non-overlapping behavioural subclasses operating at shorter timescales)? Are individuals consistent not only in constituent behaviours but also in the transition rates among behaviours? Applied specifically to the study of individual behavioural variation across development, differences in individual-level behavioural plasticity can be quantified as changes to the structure of behavioural repertoires (i.e. behavioural transition matrices). Many state-switching models can also investigate how environmental cues influence behaviour by combining both behavioural and non-behavioural data as input [148] (see §2*a*, ‘Measurement innovations’). In total, these tools bring us closer to understanding the development of entire behavioural repertoires, the ‘architecture’ of behaviour throughout development and the factors that influence individual variation in developmental trajectories.

### (c) Information integration across development

Key to understanding behavioural development is understanding the cues to which developing organisms respond, how these cues are integrated with previous information available to animals, and how specific sequences of cues shape behavioural change [111,149–152]. In particular, Bayesian updating models have been very successful at describing behavioural development [111]. Briefly, Bayesian updating as it applies to developing animals is an information theoretic method for modelling an animal's informational state (and consequently, optimal behavioural responses), given its prior information (based, for example, on evolutionary history or epigenetically inherited information [153,154]) combined with incoming, often imperfect, information through its current experience (or a sequence of experiences) [155]. While there are some empirical findings that corroborate predictions from Bayesian models of behavioural development [156–158], in general, empirical tests of these predictions remain relatively rare [111]. We believe that new experimental possibilities associated with high-resolution behavioural tracking—allowing us to track the unfolding of behaviour under different cue regimes over prolonged periods of time—put us in the position to test many of the untested predictions of Bayesian models of development.

The key predictions in Bayesian models of development relate (i) the structure of environmental fluctuations and (ii) the availability and quality of information to the degree and duration of behavioural plasticity [111,159]. As long as the environment does not change and animals receive a steady stream of informative cues throughout development, the observed degree of behavioural plasticity at an individual level (and, in some cases, behavioural diversity at a population level) is predicted to decrease with age [107,108,136,160]. If, however, reliable cues are rare, if these reliabilities change through time, or if the environment fluctuates through time, periods of plasticity throughout development may be extended [109,110,160,161]. Likewise, cues received late in life might not elicit strong behavioural responses because they may be perceived to be associated with rare events or there may not be sufficient time left in life to capitalize on any benefits conferred through altering one's phenotype [162]. Experimenters could manipulate ‘priors’ (e.g. by manipulating parental experience, or sampling from evolutionarily divergent populations), the structure of environmental fluctuations, and/or the reliability of cues during development and then follow behavioural change over development. This sort of approach could produce large enough datasets to estimate the entire developmental time course of changes in behavioural plasticity at an individual level, offering direct tests of many of the core predictions stemming from Bayesian models of behavioural development.

## 4. Limitations and future challenges

Big behavioural data are powerful tools with great potential to test fundamental open questions in behavioural development in the context of ecology and evolution (box 1). The wealth of data that high-resolution tracking provides, however, is limited in its scope to advance our knowledge of behavioural development without the idea-rich scaffolding that theory and hypothesis generation provide [163,164]. Thus, throughout this review, we have paid particular attention (see §3, ‘Using big behavioural data to uncover the principles governing behavioural development’) to how big data tools can both test and advance current theory in this field. Nevertheless, limitations remain that researchers must confront.

Box 1. **Representative major open questions in behavioural development and their big data approaches.**

**Behaviour–state connections**

*Approach:* Detect presence of dynamic feedbacks between behaviour and state (i.e. bidirectional state–behaviour linkages); quantify both the strength and direction of state–behaviour feedbacks through development.

**Q1** What are the relative rates of change in behaviour and state during development and what are the dynamic consequences when they are linked?
**Q2** How common are state–behaviour feedbacks?
**Q3** How is the strength and direction of feedbacks affected by certain ecological conditions/selective environments?
**Q4** Are feedbacks more important in shaping developmental trajectories in particular developmental stages?

**Early-life effects and behavioural transitions**

*Approach:* Measure behavioural–experiential trajectories continuously throughout life; quantify fine-grained transitions between behavioural classes throughout development.

**Q5** What are the behavioural–experiential mechanisms linking early-life experiences and long-term behavioural outcomes?
**Q6** When and to what extent are behavioural patterns throughout development path-dependent?
**Q7** To what extent are behaviours that operate at different timescales structured hierarchically?
**Q8** Do individuals differ in their behavioural transition rates? Is this an ecologically important axis of behavioural variation?

**Information integration across development**

*Approach:* Pair high-resolution behavioural tracking data with similarly resolved monitoring of environmental cues.

**Q9** How are developmental responses to recent cues moderated by the specific sequences of past cues?
**Q10** How does behavioural development relate to the structure of environmental fluctuations?
**Q11** How does the availability and quality of information affect behavioural development?

First, linking high-resolution behavioural tracking to metrics requiring terminal sampling (e.g. gene expression changes in the brain or experience-dependent epigenetic changes across development) will require creative experimental design and/or the development of new technologies (e.g. multi-electrode arrays or wireless neurotelemetry [165]). Further challenges arise when considering the combination of high-resolution behavioural and environmental time series. In some cases, salient environmental changes that have dramatic effects on behavioural development may occur only rarely (e.g. once or twice over the course of an individual's development), limiting the ability of researchers to estimate their effects. These types of scenarios would make studies that look for correlations among environmental metrics from passive sensors and high-resolution behavioural data difficult; on the other hand, they may offer great opportunities for experimentally manipulating candidate environmental variables/stressors (their timing or intensity, for example) while tracking behavioural development. Next, while methodological advances allowing the collection of temporally rich behavioural data across development vastly reduce the amount of work effort per datapoint, a fundamental limitation that will always remain is the relative lifespan (in particular, the duration of development) of research animals. For species with long developmental periods, there are inherent challenges to collecting near-continuous developmental data. This might be partially addressed, for example, by subsampling populations of longer-lived species by age class and conducting behavioural observations over shorter periods of development (relative to that species) but for individuals spread across age classes in order to get a fuller picture of development in the aggregate (e.g. combine approaches 4 and 5 in table 1). These and similar sorts of trade-offs will exist in most experiments in which researchers desire to test the whole of behavioural development, but the ever-growing technological advances in this field are also likely to continue to rebalance these trade-offs in favour of ever-richer behavioural developmental datasets across species.

## 5. Concluding remarks

For the last three-quarters of a century, a steady stream of technological advancement has allowed the discovery of new behaviours as well as innovations in the quantification of previously observed behaviours (e.g. audio recording of echolocation in bats [166] and cetaceans [167], spectrographic analysis of birdsong [168], high-speed cameras revealing feeding innovations [169,170], automatic sensing and social network analysis uncovering novel social and collective behaviours [65], machine learning algorithms as behavioural classifiers [89]). In turn, these technological advances in quantifying behaviour have been productively applied to understanding both the proximate and evolutionary causes and consequences of behaviour [171], helping to transform the field of animal behaviour from a largely descriptive science to an analytical and theoretical one [172]. Now, with the widening application of state-of-the-art tracking technologies, the time is ripe to continue in this tradition by applying these tools to major open questions in one of behavioural ecology's foundational research cores: the ontogeny of behaviour.

## Data Availability

This article has no additional data.

## References

[1] Nath T, Mathis A, Chen AC, Patel A, Bethge M, Mathis MW. 2019 Using DeepLabCut for 3D markerless pose estimation across species and behaviors. Nat. Protoc. **14**, 2152-2176. (10.1038/s41596-019-0176-0) 31227823

[2] Lauer J et al. 2022 Multi-animal pose estimation, identification and tracking with DeepLabCut. Nat. Methods **19**, 496-504. (10.1038/s41592-022-01443-0) 35414125 PMC9007739

[3] Francisco FA, Nührenberg P, Jordan A. 2020 High-resolution, non-invasive animal tracking and reconstruction of local environment in aquatic ecosystems. Mov. Ecol. **8**, 27. (10.1186/s40462-020-00214-w) 32582448 PMC7310323

[4] Alarcón-Nieto G, Graving JM, Klarevas-Irby JA, Maldonado-Chaparro AA, Mueller I, Farine DR. 2018 An automated barcode tracking system for behavioural studies in birds. Methods Ecol. Evol. **9**, 1536-1547. (10.1111/2041-210X.13005)

[5] Haalck L, Mangan M, Webb B, Risse B. 2020 Towards image-based animal tracking in natural environments using a freely moving camera. J. Neurosci. Methods **330**, 108455. (10.1016/j.jneumeth.2019.108455) 31739118

[6] Pérez-Escudero A, Vicente-Page J, Hinz RC, Arganda S, de Polavieja GG. 2014 idTracker: tracking individuals in a group by automatic identification of unmarked animals. Nat. Methods **11**, 743-748. (10.1038/nmeth.2994) 24880877

[7] McDiarmid TA, Yu AJ, Rankin CH. 2018 Beyond the response—high throughput behavioral analyses to link genome to phenome in *Caenorhabditis elegans*. Genes Brain Behav. **17**, e12437. (10.1111/gbb.12437) 29124896

[8] Jones BM et al. 2020 Individual differences in honey bee behavior enabled by plasticity in brain gene regulatory networks. eLife **9**, e62850. (10.7554/eLife.62850) 33350385 PMC7755388

[9] Franchini P, Irisarri I, Fudickar A, Schmidt A, Meyer A, Wikelski M, Partecke J. 2017 Animal tracking meets migration genomics: transcriptomic analysis of a partially migratory bird species. Mol. Ecol. **26**, 3204-3216. (10.1111/mec.14108) 28316119

[10] von Ziegler L, Sturman O, Bohacek J. 2020 Big behavior: challenges and opportunities in a new era of deep behavior profiling. Neuropsychopharmacology **46** 33-44. (10.1038/s41386-020-0751-7) 32599604 PMC7688651

[11] Gomez-Marin A, Ghazanfar AA. 2019 The life of behavior. Neuron **104**, 25-36. (10.1016/j.neuron.2019.09.017) 31600513 PMC6873815

[12] Datta SR, Anderson DJ, Branson K, Perona P, Leifer A. 2019 Computational neuroethology: a call to action. Neuron **104**, 11-24. (10.1016/j.neuron.2019.09.038) 31600508 PMC6981239

[13] Ghosh M, Rihel J. 2020 Hierarchical compression reveals sub-second to day-long structure in larval zebrafish behavior. eNeuro **7**, ENEURO.0408-19.2020. (10.1523/ENEURO.0408-19.2020) PMC7405074 32241874

[14] Dunn TW et al. 2016 Brain-wide mapping of neural activity controlling zebrafish exploratory locomotion. eLife **5**, e12741. (10.7554/eLife.12741) 27003593 PMC4841782

[15] Gomez-Marin A, Paton JJ, Kampff AR, Costa RM, Mainen ZF. 2014 Big behavioral data: psychology, ethology and the foundations of neuroscience. Nat. Neurosci. **17**, 1455–1462. (10.1038/nn.3812) 25349912

[16] Strandburg-Peshkin A, Farine DR, Couzin ID, Crofoot MC. 2015 Shared decision-making drives collective movement in wild baboons. Science **348**, 1358-1361. (10.1126/science.aaa5099) 26089514 PMC4801504

[17] Nagy M, Vasarhelyi G, Pettit B, Roberts-Mariani I, Vicsek T, Biro D. 2013 Context-dependent hierarchies in pigeons. Proc. Natl Acad. Sci. USA **110**, 13 049-13 054. (10.1073/pnas.1305552110) PMC3740899 23878247

[18] Jolles JW, Boogert NJ, Sridhar VH, Couzin ID, Manica A. 2017 Consistent Individual differences drive collective behavior and group functioning of schooling fish. Curr. Biol. **27**, 2862-2868.e7. (10.1016/j.cub.2017.08.004) 28889975 PMC5628957

[19] Jolles JW, Laskowski KL, Boogert NJ, Manica A. 2018 Repeatable group differences in the collective behaviour of stickleback shoals across ecological contexts. Proc. R. Soc. B. **285**, 20172629. (10.1098/rspb.2017.2629) PMC5829202 29436496

[20] Westley PAH, Berdahl AM, Torney CJ, Biro D. 2018 Collective movement in ecology: from emerging technologies to conservation and management. Phil. Trans. R. Soc. B **373**, 20170004. (10.1098/rstb.2017.0004) 29581389 PMC5882974

[21] Wijeyakulasuriya DA, Eisenhauer EW, Shaby BA, Hanks EM. 2020 Machine learning for modeling animal movement. PLoS ONE **15**, e0235750. (10.1371/journal.pone.0235750) 32716917 PMC7384613

[22] Dell AI et al. 2014 Automated image-based tracking and its application in ecology. Trends Ecol. Evol. **29**, 417-428. (10.1016/j.tree.2014.05.004) 24908439

[23] Nathan R et al. 2022 Big-data approaches lead to an increased understanding of the ecology of animal movement. Science **375**, eabg1780. (10.1126/science.abg1780) 35175823

[24] Boogert NJ, Farine DR, Spencer KA. 2014 Developmental stress predicts social network position. Biol. Lett. **10**, 20140561. (10.1098/rsbl.2014.0561) 25354917 PMC4272205

[25] Bateson PPG, Jaeckel JB. 1976 Chicks’ preferences for familiar and novel conspicuous objects after different periods of exposure. Anim. Behav. **24**, 386-390. (10.1016/S0003-3472(76)80048-6)

[26] Schürch R, Heg D. 2010 Life history and behavioral type in the highly social cichlid *Neolamprologus pulcher*. Behav. Ecol. **21**, 588-598. (10.1093/beheco/arq024)

[27] Bell AM, Stamps JA. 2004 Development of behavioural differences between individuals and populations of sticklebacks, *Gasterosteus aculeatus*. Anim. Behav. **68**, 1339-1348. (10.1016/j.anbehav.2004.05.007)

[28] Neave HW, Costa JHC, Weary DM, von Keyserlingk MAG. 2020 Long-term consistency of personality traits of cattle. R. Soc. Open Sci. **7**, 191849. (10.1098/rsos.191849) 32257341 PMC7062087

[29] Kok EMA, Burant JB, Dekinga A, Manche P, Saintonge D, Piersma T, Mathot KJ. 2019 Within-individual canalization contributes to age-related increases in trait repeatability: a longitudinal experiment in red knots. Am. Nat. **194**, 455-469. (10.1086/704593) 31490730

[30] Edenbrow M, Croft DP. 2011 Behavioural types and life history strategies during ontogeny in the mangrove killifish, *Kryptolebias marmoratus*. Anim. Behav. **82**, 731-741. (10.1016/j.anbehav.2011.07.003)

[31] DiRienzo N, Pruitt JN, Hedrick AV. 2012 Juvenile exposure to acoustic sexual signals from conspecifics alters growth trajectory and an adult personality trait. Anim. Behav. **84**, 861-868. (10.1016/j.anbehav.2012.07.007)

[32] Ehlman SM, Sandkam BA, Breden F, Sih A. 2015 Developmental plasticity in vision and behavior may help guppies overcome increased turbidity. J. Comp. Physiol. A **201**, 1125-1135. (10.1007/s00359-015-1041-4) 26427995

[33] Urszán TJ, Garamszegi LZ, Nagy G, Hettyey A, Török J, Herczeg G. 2015 No personality without experience? A test on *Rana dalmatina* tadpoles. Ecol. Evol. **5**, 5847-5856. (10.1002/ece3.1804) 26811759 PMC4717344

[34] Balsam JS, Stevenson PA. 2021 Agonistic experience during development establishes inter-individual differences in approach-avoidance behaviour of crickets. Scient. Rep. **11**, 16702. (10.1038/s41598-021-96201-1) PMC8371163 34404861

[35] Fischer S, Bohn L, Oberhummer E, Nyman C, Taborsky B. 2017 Divergence of developmental trajectories is triggered interactively by early social and ecological experience in a cooperative breeder. Proc. Natl Acad. Sci. USA **114**, E9300-E9307. (10.1073/pnas.1705934114) 29078289 PMC5676887

[36] Gottlieb G. 1961 Developmental age as a baseline for determination of the critical period in imprinting. J. Comp. Physiol. Psychol. **54**, 422-427. (10.1037/h0049127) 13707440

[37] Petelle MB, McCoy DE, Alejandro V, Martin JG, Blumstein DT. 2013 Development of boldness and docility in yellow-bellied marmots. Anim. Behav. **86**, 1147-1154. (10.1016/j.anbehav.2013.09.016)

[38] Wuerz Y, Krüger O. 2015 Personality over ontogeny in zebra finches: long-term repeatable traits but unstable behavioural syndromes. Front. Zool. **12**, S9. (10.1186/1742-9994-12-S1-S9) 26813709 PMC4722341

[39] Smith ML, Davidson JD, Wild B, Dormagen DM, Landgraf T, Couzin ID. 2022 Behavioral variation across the days and lives of honey bees. iScience **25**, 104842. (10.1016/j.isci.2022.104842) 36039297 PMC9418442

[40] Laskowski KL, Bierbach D, Jolles JW, Doran C, Wolf M. 2022 The emergence and development of behavioral individuality in clonal fish. Nat. Commun. **13**, 6419. (10.1038/s41467-022-34113-y) 36307437 PMC9616841

[41] Kennedy A. 2022 The what, how, and why of naturalistic behavior. Curr. Opin. Neurobiol. **74**, 102549. (10.1016/j.conb.2022.102549) 35537373 PMC9273162

[42] Brown AEX, de Bivort B. 2018 Ethology as a physical science. Nat. Phys. **14**, 653-657. (10.1038/s41567-018-0093-0)

[43] Berman GJ, Choi DM, Bialek W, Shaevitz JW. 2014 Mapping the stereotyped behaviour of freely moving fruit flies. J. R. Soc. Interface **11**, 20140672. (10.1098/rsif.2014.0672) 25142523 PMC4233753

[44] Berman GJ, Bialek W, Shaevitz JW. 2016 Predictability and hierarchy in *Drosophila* behavior. Proc. Natl Acad. Sci. USA **113**, 11 943-11 948. (10.1073/pnas.1607601113) PMC5081631 27702892

[45] Wiltschko AB, Johnson MJ, Iurilli G, Peterson RE, Katon JM, Pashkovski SL, Abraira VE, Adams RP, Datta SR. 2015 Mapping sub-second structure in mouse behavior. Neuron **88**, 1121-1135. (10.1016/j.neuron.2015.11.031) 26687221 PMC4708087

[46] Hernández DG, Rivera C, Cande J, Zhou B, Stern DL, Berman GJ. 2021 A framework for studying behavioral evolution by reconstructing ancestral repertoires. eLife **10**, e61806. (10.7554/eLife.61806) 34473052 PMC8445618

[47] Pereira TD et al. 2022 SLEAP: a deep learning system for multi-animal pose tracking. Nat. Methods **19**, 486-495. (10.1038/s41592-022-01426-1) 35379947 PMC9007740

[48] Walter T, Couzin ID. 2021 TRex, a fast multi-animal tracking system with markerless identification, and 2D estimation of posture and visual fields. eLife **10**, e64000. (10.7554/eLife.64000) 33634789 PMC8096434

[49] Rodriguez A, Zhang H, Klaminder J, Brodin T, Andersson PL, Andersson M. 2018 *ToxTrac*: a fast and robust software for tracking organisms. Methods Ecol. Evol. **9**, 460-464. (10.1111/2041-210X.12874)

[50] Jolles JW. 2021 Broad-scale applications of the Raspberry Pi: a review and guide for biologists. Methods Ecol. Evol. **12**, 1562-1579. (10.1111/2041-210X.13652)

[51] Mathis MW, Mathis A. 2020 Deep learning tools for the measurement of animal behavior in neuroscience. Curr. Opin. Neurobiol. **60**, 1-11. (10.1016/j.conb.2019.10.008) 31791006

[52] Pereira TD, Shaevitz JW, Murthy M. 2020 Quantifying behavior to understand the brain. Nat. Neurosci. **23**, 1537-1549. (10.1038/s41593-020-00734-z) 33169033 PMC7780298

[53] Monkman GG, Hyder K, Kaiser MJ, Vidal FP. 2019 Using machine vision to estimate fish length from images using regional convolutional neural networks. Methods Ecol. Evol. **10**, 2045-2056. (10.1111/2041-210X.13282)

[54] Williams HJ et al. 2020 Optimizing the use of biologgers for movement ecology research. J. Anim. Ecol. **89**, 186-206. (10.1111/1365-2656.13094) 31424571 PMC7041970

[55] Chmura HE, Glass TW, Williams CT. 2018 Biologging physiological and ecological responses to climatic variation: new tools for the climate change era. Front. Ecol. Evol. **6**, 92. (10.3389/fevo.2018.00092)

[56] Goldbogen JA et al. 2019 Extreme bradycardia and tachycardia in the world's largest animal. Proc. Natl Acad. Sci. USA **116**, 25 329-25 332. (10.1073/pnas.1914273116) PMC6911174 31767746

[57] Aoki K, Watanabe Y, Inamori D, Funasaka N, Sakamoto KQ. 2021 Towards non-invasive heart rate monitoring in free-ranging cetaceans: a unipolar suction cup tag measured the heart rate of trained Risso's dolphins. Phil. Trans. R. Soc. B **376**, 20200225. (10.1098/rstb.2020.0225) 34176321 PMC8243410

[58] Reher S, Dausmann KH. 2021 Tropical bats counter heat by combining torpor with adaptive hyperthermia. Proc. R. Soc. B **288**, 20202059. (10.1098/rspb.2020.2059) PMC7892405 33434466

[59] Cilulko J, Janiszewski P, Bogdaszewski M, Szczygielska E. 2013 Infrared thermal imaging in studies of wild animals. Eur. J. Wildl. Res. **59**, 17-23. (10.1007/s10344-012-0688-1)

[60] Demartsev V, Manser MB, Tattersall GJ. 2022 Vocalization-associated respiration patterns: thermography-based monitoring and detection of preparation for calling. J. Exp. Biol. **225**, jeb243474. (10.1242/jeb.243474) 35142353 PMC8976942

[61] Andresen N, Wöllhaf M, Hohlbaum K, Lewejohann L, Hellwich O, Thöne-Reineke C, Belik V. 2020 Towards a fully automated surveillance of well-being status in laboratory mice using deep learning: starting with facial expression analysis. PLoS ONE **15**, e0228059. (10.1371/journal.pone.0228059) 32294094 PMC7159220

[62] Chen C, Zhu W, Steibel J, Siegford J, Wurtz K, Han J, Norton T. 2020 Recognition of aggressive episodes of pigs based on convolutional neural network and long short-term memory. Comput. Electron. Agric. **169**, 105166. (10.1016/j.compag.2019.105166)

[63] Calhoun AJ, Pillow JW, Murthy M. 2019 Unsupervised identification of the internal states that shape natural behavior. Nat. Neurosci. **22**, 2040-2049. (10.1038/s41593-019-0533-x) 31768056 PMC7819718

[64] Briefer EF et al. 2022 Classification of pig calls produced from birth to slaughter according to their emotional valence and context of production. Scient. Rep. **12**, 3409. (10.1038/s41598-022-07174-8) PMC8901661 35256620

[65] Smith JE, Pinter-Wollman N. 2021 Observing the unwatchable: integrating automated sensing, naturalistic observations and animal social network analysis in the age of big data. J. Anim. Ecol. **90**, 62-75. (10.1111/1365-2656.13362) 33020914

[66] Frankenhuis WE, Nettle D, Dall SRX. 2019 A case for environmental statistics of early-life effects. Phil. Trans. R. Soc. B **374**, 20180110. (10.1098/rstb.2018.0110) 30966883 PMC6460088

[67] Hawkins WD, DuRant SE. 2020 Applications of machine learning in behavioral ecology: quantifying avian incubation behavior and nest conditions in relation to environmental temperature. PLoS ONE **15**, e0236925. (10.1371/journal.pone.0236925) 32857761 PMC7454991

[68] Levitis DA, Lidicker Jr WZ, Freund G. 2009 Behavioural biologists do not agree on what constitutes behaviour. Anim. Behav. **78**, 103-110. (10.1016/j.anbehav.2009.03.018) 20160973 PMC2760923

[69] Carter AJ, Feeney WE, Marshall HH, Cowlishaw G, Heinsohn R. 2013 Animal personality: what are behavioural ecologists measuring? Biol. Rev. Camb. Phil. Soc. **88**, 465-475. (10.1111/brv.12007) 23253069

[70] Bohnslav JP et al. 2021 DeepEthogram, a machine learning pipeline for supervised behavior classification from raw pixels. eLife **10**, e63377. (10.7554/eLife.63377) 34473051 PMC8455138

[71] Kain J, Stokes C, Gaudry Q, Song X, Foley J, Wilson R, de Bivort B. 2013 Leg-tracking and automated behavioural classification in *Drosophila*. Nat. Commun. **4**, 1910. (10.1038/ncomms2908) 23715269 PMC3674277

[72] Budaev SV. 2010 Using principal components and factor analysis in animal behaviour research: caveats and guidelines. Ethology **116**, 472-480. (10.1111/j.1439-0310.2010.01758.x)

[73] Heerah K, Woillez M, Fablet R, Garren F, Martin S, De Pontual H. 2017 Coupling spectral analysis and hidden Markov models for the segmentation of behavioural patterns. Mov. Ecol. **5**, 20. (10.1186/s40462-017-0111-3) 28944062 PMC5609058

[74] van der Maaten L, Hinton G. 2008 Visualizing data using t-SNE. J. Machine Learn. Res. **9**, 2579-2605.

[75] McInnes L, Healy J. 2018 UMAP: Uniform Manifold Approximation and Projection for Dimension Reduction. *arXiv*, 1802.03426. (10.48550/arXiv.1802.03426)

[76] Overman KE, Choi DM, Leung K, Shaevitz JW, Berman GJ. 2022 Measuring the repertoire of age-related behavioral changes in *Drosophila melanogaster*. PLoS Comput. Biol. **18**, e1009867. (10.1371/journal.pcbi.1009867) 35202388 PMC8903287

[77] Huang K et al. 2021 A hierarchical 3D-motion learning framework for animal spontaneous behavior mapping. Nat. Commun. **12**, 2784. (10.1038/s41467-021-22970-y) 33986265 PMC8119960

[78] Klibaite U, Shaevitz JW. 2020 Paired fruit flies synchronize behavior: uncovering social interactions in *Drosophila melanogaster*. PLoS Comput. Biol. **16**, e1008230. (10.1371/journal.pcbi.1008230) 33021989 PMC7567355

[79] Valletta JJ, Torney C, Kings M, Thornton A, Madden J. 2017 Applications of machine learning in animal behaviour studies. Anim. Behav. **124**, 203-220. (10.1016/j.anbehav.2016.12.005)

[80] Todd JG, Kain JS, de Bivort BL. 2017 Systematic exploration of unsupervised methods for mapping behavior. Phys. Biol. **14**, 015002. (10.1088/1478-3975/14/1/015002) 28166059

[81] Goldberg LR. 1993 The structure of phenotypic personality traits. Am. Psychol. **48**, 26-34. (10.1037/0003-066X.48.1.26) 8427480

[82] Heyser CJ, Chemero A. 2012 Novel object exploration in mice: not all objects are created equal. Behav. Process. **89**, 232-238. (10.1016/j.beproc.2011.12.004) 22183090

[83] Kaiser MI, Müller C. 2021 What is an animal personality? Biol. Philosophy **36**, 1. (10.1007/s10539-020-09776-w)

[84] Groothuis TGG, Trillmich F. 2011 Unfolding personalities: the importance of studying ontogeny. Dev. Psychobiol. **53**, 641-655. (10.1002/dev.20574) 21866544

[85] White SJ, Pascall DJ, Wilson AJ. 2020 Towards a comparative approach to the structure of animal personality variation. Behav. Ecol. **31**, 340-351. (10.1093/beheco/arz198) 32210524 PMC7083098

[86] Carter AJ, Feeney WE. 2012 Taking a comparative approach: analysing personality as a multivariate behavioural response across species. PLoS ONE **7**, e42440. (10.1371/journal.pone.0042440) 22860126 PMC3409165

[87] Dingemanse NJ, Dochtermann N, Wright J. 2010 A method for exploring the structure of behavioural syndromes to allow formal comparison within and between data sets. Anim. Behav. **79**, 439-450. (10.1016/j.anbehav.2009.11.024)

[88] Marques JC, Lackner S, Félix R, Orger MB. 2018 Structure of the zebrafish locomotor repertoire revealed with unsupervised behavioral clustering. Curr. Biol. **28**, 181-195.e5. (10.1016/j.cub.2017.12.002) 29307558

[89] Clemens J, Coen P, Roemschied FA, Pereira TD, Mazumder D, Aldarondo DE, Pacheco DA, Murthy M. 2018 Discovery of a new song mode in *Drosophila* reveals hidden structure in the sensory and neural drivers of behavior. Curr. Biol. **28**, 2400-2412.e6. (10.1016/j.cub.2018.06.011) 30057309 PMC6830513

[90] Tinbergen N. 1951 The study of instinct. Oxford, UK: Clarendon Press.

[91] Dawkins R. 1976 Hierarchical organisation: a candidate principle for ethology. In Growing points in ethology (eds PPG Bateson, RA Hinde), pp. 7–54. Cambridge, UK: Cambridge University Press.

[92] Conners MG, Michelot T, Heywood EI, Orben RA, Phillips RA, Vyssotski AL, Shaffer SA, Thorne LH. 2021 Hidden Markov models identify major movement modes in accelerometer and magnetometer data from four albatross species. Mov. Ecol. **9**, 7. (10.1186/s40462-021-00243-z) 33618773 PMC7901071

[93] Leos-Barajas V, Gangloff EJ, Adam T, Langrock R, van Beest FM, Nabe-Nielsen J, Morales JM. 2017 Multi-scale modeling of animal movement and general behavior data using hidden Markov models with hierarchical structures. J. Agric. Biol. Environ. Stat. **22**, 232-248. (10.1007/s13253-017-0282-9)

[94] Adam T, Griffiths CA, Leos-Barajas V, Meese EN, Lowe CG, Blackwell PG, Righton D, Langrock R. 2019 Joint modelling of multi-scale animal movement data using hierarchical hidden Markov models. Methods Ecol. Evol. **10**, 1536-1550. (10.1111/2041-210X.13241)

[95] Montiglio PO, Ferrari C, Réale D. 2013 Social niche specialization under constraints: personality, social interactions and environmental heterogeneity. Phil. Trans. R. Soc. B **368**, 20120343. (10.1098/rstb.2012.0343) 23569291 PMC3638446

[96] Bergmüller R, Taborsky M. 2007 Adaptive behavioural syndromes due to strategic niche specialization. BMC Ecol. **7**, 12. (10.1186/1472-6785-7-12) 17935618 PMC2104524

[97] Nonacs P. 2001 State dependent behavior and the marginal value theorem. Behav. Ecol. **12**, 71-83. (10.1093/oxfordjournals.beheco.a000381)

[98] Rands SA, Pettifor RA, Rowcliffe JM, Cowlishaw G. 2004 State-dependent foraging rules for social animals in selfish herds. Proc. R. Soc. Lond. B **271**, 2613-2620. (10.1098/rspb.2004.2906) PMC1691894 15615688

[99] Ehlman SM, Trimmer PC, Sih A. 2019 Prey responses to exotic predators: effects of old risks and new cues. Am. Nat. **193**, 575-587. (10.1086/702252) 30912973

[100] Olsson O, Brown JS, Smith HG. 2002 Long- and short-term state-dependent foraging under predation risk: an indication of habitat quality. Anim. Behav. **63**, 981-989. (10.1006/anbe.2001.1985)

[101] Tigreros N, Wang EH, Thaler JS. 2018 Prey nutritional state drives divergent behavioural and physiological responses to predation risk. Funct. Ecol. **77**, 1116. (10.1111/1365-2435.13046)

[102] Budaev S, Jørgensen C, Mangel M, Eliassen S, Giske J. 2019 Decision-making from the animal perspective: bridging ecology and subjective cognition. Front. Ecol. Evol. **7**, 164. (10.3389/fevo.2019.00164)

[103] Giske J, Eliassen S, Fiksen Ø, Jakobsen PJ, Aksnes DL, Jørgensen C, Mangel M. 2013 Effects of the emotion system on adaptive behavior. Am. Nat. **182**, 689-703. (10.1086/673533) 24231532

[104] McHuron EA, Costa DP, Schwarz L, Mangel M. 2017 State-dependent behavioural theory for assessing the fitness consequences of anthropogenic disturbance on capital and income breeders. Methods Ecol. Evol. **8**, 552-560. (10.1111/2041-210X.12701)

[105] Trimmer PC, Ehlman SM, Sih A. 2017 Predicting behavioural responses to novel organisms: state-dependent detection theory. Proc. R. Soc. B **284**, 20162108. (10.1098/rspb.2016.2108) PMC5310033 28100814

[106] Clark CW, Mangel M. 2000 Dynamic state variable models in ecology: methods and applications. Oxford, UK: Oxford University Press.

[107] Frankenhuis WE, Panchanathan K. 2011 Balancing sampling and specialization: an adaptationist model of incremental development. Proc. R. Soc. B **278**, 3558-3565. (10.1098/rspb.2011.0055) PMC3189362 21490018

[108] Panchanathan K, Frankenhuis WE. 2016 The evolution of sensitive periods in a model of incremental development. Proc. R. Soc. B **283**, 20152439. (10.1098/rspb.2015.2439) PMC4795025 26817766

[109] Walasek N, Frankenhuis WE, Panchanathan K. 2021 An evolutionary model of sensitive periods when the reliability of cues varies across ontogeny. Behav. Ecol. **113**, 101-114. (10.1093/beheco/arab113) PMC8857937 35197808

[110] Walasek N, Frankenhuis WE, Panchanathan K. 2022 Sensitive periods, but not critical periods, evolve in a fluctuating environment: a model of incremental development. Proc. R. Soc. B. **289**, 20212623. (10.1098/rspb.2021.2623) PMC8848242 35168396

[111] Stamps JA, Frankenhuis WE. 2016 Bayesian models of development. Trends Ecol. Evol. **31**, 260-268. (10.1016/j.tree.2016.01.012) 26896042

[112] Houston AI, McNamara JM. 1999 Models of adaptive behaviour: an approach based on state. Cambridge, UK: Cambridge University Press.

[113] Sih A, Mathot KJ, Moirón M, Montiglio PO, Wolf M, Dingemanse NJ. 2015 Animal personality and state–behaviour feedbacks: a review and guide for empiricists. Trends Ecol. Evol. **30**, 50-60. (10.1016/j.tree.2014.11.004) 25498413

[114] Taborsky B. 2021 A positive feedback loop between sociality and social competence. Ethology **127**, 774-789. (10.1111/eth.13201)

[115] Ehlman SM, Scherer U, Wolf M. 2022 Developmental feedbacks and the emergence of individuality. R. Soc. Open Sci. **9**, 221189. (10.1098/rsos.221189) 36465682 PMC9709565

[116] MacGregor HEA, Cottage A, Ioannou CC. 2021 Suppression of personality variation in boldness during foraging in three-spined sticklebacks. Behav. Ecol. Sociobiol. **75**, 71. (10.1007/s00265-021-03007-2)

[117] Mathot KJ, Dekinga A, Piersma T. 2017 An experimental test of state–behaviour feedbacks: gizzard mass and foraging behaviour in red knots. Funct. Ecol. **31**, 1111-1121. (10.1111/1365-2435.12827)

[118] Petelle MB, Martin JGA, Blumstein DT. 2019 Mixed support for state maintaining risky personality traits in yellow-bellied marmots. Anim. Behav. **150**, 177-188. (10.1016/j.anbehav.2019.02.008)

[119] Laskowski KL, Chang CC, Sheehy K, Aguiñaga J. 2022 Consistent individual behavioral variation: what do we know and where are we going? Annu. Rev. Ecol. Evol. Syst. **53**, 161-182. (10.1146/annurev-ecolsys-102220-011451)

[120] Clark CW. 1994 Antipredator behavior and the asset-protection principle. Behav. Ecol. **5**, 159-170. (10.1093/beheco/5.2.159)

[121] Luttbeg B, Sih A. 2010 Risk, resources and state-dependent adaptive behavioural syndromes. Phil. Trans. R. Soc. B **365**, 3977-3990. (10.1098/rstb.2010.0207) 21078650 PMC2992746

[122] Wolf M, van Doorn GS, Leimar O, Weissing FJ. 2007 Life-history trade-offs favour the evolution of animal personalities. Nature **447**, 581-584. (10.1038/nature05835) 17538618

[123] Ferrari MCO, Brown GE, Bortolotti GR, Chivers DP. 2010 Linking predator risk and uncertainty to adaptive forgetting: a theoretical framework and empirical test using tadpoles. Proc. R. Soc. B **277**, 2205-2210. (10.1098/rspb.2009.2117) PMC2880143 20236976

[124] Stamps J, Groothuis T. 2010 The development of animal personality: relevance, concepts and perspectives. Biol. Rev. **85**, 301-325. (10.1111/j.1469-185X.2009.00103.x) 19961473

[125] Carlson BA. 2017 Early life experiences have complex and long-lasting effects on behavior. Proc. Natl Acad. Sci. USA **114**, 11 571-11 573. (10.1073/pnas.1716037114) 29078413 PMC5676937

[126] Horn G. 1985 Memory, imprinting, and the brain. Oxford, UK: Oxford University Press.

[127] Bateson P, Horn G. 1994 Imprinting and recognition memory: a neural-net model. Anim. Behav. **48**, 695-715. (10.1006/anbe.1994.1289)

[128] Laskowski KL, Wolf M, Bierbach D. 2016 The making of winners (and losers): how early dominance interactions determine adult social structure in a clonal fish. Proc. R. Soc. B **283**, 20160183. (10.1098/rspb.2016.0183) PMC4874710 27170711

[129] Eachus H, Choi MK, Ryu S. 2021 The effects of early life stress on the brain and behaviour: insights from zebrafish models. Front. Cell Dev. Biol. **9**, 657591. (10.3389/fcell.2021.657591) 34368117 PMC8335398

[130] Langenhof MR, Komdeur J. 2018 Why and how the early-life environment affects development of coping behaviours. Behav. Ecol. Sociobiol. **72**, 34. (10.1007/s00265-018-2452-3) 29449757 PMC5805793

[131] DeWitt TJ, Sih A, Wilson DS. 1998 Costs and limits of phenotypic plasticity. Trends Ecol. Evol. **13**, 77-81. (10.1016/S0169-5347(97)01274-3) 21238209

[132] Fawcett TW, Frankenhuis WE. 2015 Adaptive explanations for sensitive windows in development. Front. Zool. **12**, S3. (10.1186/1742-9994-12-S1-S3) 26816521 PMC4722342

[133] Hayward AD, Rickard IJ, Lummaa V. 2013 Influence of early-life nutrition on mortality and reproductive success during a subsequent famine in a preindustrial population. Proc. Natl Acad. Sci. USA **110**, 13 886-13 891. (10.1073/pnas.1301817110) PMC3752237 23918366

[134] Rödel HG, von Holst D, Kraus C. 2009 Family legacies: short- and long-term fitness consequences of early-life conditions in female European rabbits. J. Anim. Ecol. **78**, 789-797. (10.1111/j.1365-2656.2009.01537.x) 19298614

[135] Marshall HH et al. 2017 Lifetime fitness consequences of early-life ecological hardship in a wild mammal population. Ecol. Evol. **7**, 1712-1724. (10.1002/ece3.2747) 28331582 PMC5355200

[136] English S, Fawcett TW, Higginson AD, Trimmer PC, Uller T. 2016 Adaptive use of information during growth can explain long-term effects of early life experiences. Am. Nat. **187**, 620-632. (10.1086/685644) 27104994

[137] Burton T, Metcalfe NB. 2014 Can environmental conditions experienced in early life influence future generations? Proc. R. Soc. B **281**, 20140311. (10.1098/rspb.2014.0311) PMC4024293 24807254

[138] Groothuis TGG, Taborsky B. 2015 Introducing biological realism into the study of developmental plasticity in behaviour. Front. Zool. **12**, S6. (10.1186/1742-9994-12-S1-S6) 26816523 PMC4722348

[139] Hoke KL, Adkins-Regan E, Bass AH, McCune AR, Wolfner MF. 2019 Co-opting evo-devo concepts for new insights into mechanisms of behavioural diversity. J. Exp. Biol. **222**, jeb190058. (10.1242/jeb.190058) 30988051 PMC6503947

[140] Salazar-Ciudad I, Jernvall J. 2005 Graduality and innovation in the evolution of complex phenotypes: insights from development. J. Exp. Zool. B Dev. Evol. **304B**, 619-631. (10.1002/jez.b.21058) 16032700

[141] Herbert-Read JE, Krause S, Morrell LJ, Schaerf TM, Krause J, Ward AJW. 2013 The role of individuality in collective group movement. Proc. R. Soc. B **280**, 20122564. (10.1098/rspb.2012.2564) PMC3574314 23222452

[142] Hansen MJ et al. 2020 Linking hunting weaponry to attack strategies in sailfish and striped marlin. Proc. R. Soc. B **287**, 20192228. (10.1098/rspb.2019.2228) PMC7003464 31937224

[143] Berman GJ. 2018 Measuring behavior across scales. BMC Biol. **16**, 23. (10.1186/s12915-018-0494-7) 29475451 PMC5824583

[144] Eyjolfsdottir E, Branson K, Yue Y, Perona P. 2016 Learning recurrent representations for hierarchical behavior modeling. *arXiv*, 1611.00094. (10.48550/arXiv.1611.00094)

[145] Katsov AY, Freifeld L, Horowitz M, Kuehn S, Clandinin TR. 2017 Dynamic structure of locomotor behavior in walking fruit flies. eLife **6**, e26410. (10.7554/eLife.26410) 28742018 PMC5526672

[146] Ahamed T, Costa AC, Stephens GJ. 2021 Capturing the continuous complexity of behaviour in *Caenorhabditis elegans*. Nat. Phys. **17**, 275-283. (10.1038/s41567-020-01036-8)

[147] Stephens GJ, de Mesquita MB, Ryu WS, Bialek W. 2011 Emergence of long timescales and stereotyped behaviors in *Caenorhabditis elegans*. Proc. Natl Acad. Sci. USA **108**, 7286-7289. (10.1073/pnas.1007868108) 21502536 PMC3088607

[148] Bestley S, Jonsen ID, Hindell MA, Guinet C, Charrassin JB. 2013 Integrative modelling of animal movement: incorporating *in situ* habitat and behavioural information for a migratory marine predator. Proc. R. Soc. B **280**, 20122262. (10.1098/rspb.2012.2262) PMC3574443 23135676

[149] Fawcett TW, Johnstone RA. 2003 Optimal assessment of multiple cues. Proc. R. Soc. B **270**, 1637-1643. (10.1098/rspb.2003.2328) PMC1691413 12908986

[150] Munoz NE, Blumstein DT. 2012 Multisensory perception in uncertain environments. Behav. Ecol. **23**, 457-462. (10.1093/beheco/arr220)

[151] Winkler DW et al. 2014 Cues, strategies, and outcomes: how migrating vertebrates track environmental change. Mov. Ecol. **2**, 10. (10.1186/2051-3933-2-10)

[152] Chmura HE, Kharouba HM, Ashander J, Ehlman SM, Rivest EB, Yang LH. 2018 The mechanisms of phenology: the patterns and processes of phenological shifts. Ecol. Monogr. **89**, e01337. (10.1002/ecm.1337)

[153] Dall SRX, McNamara JM, Leimar O. 2015 Genes as cues: phenotypic integration of genetic and epigenetic information from a Darwinian perspective. Trends Ecol. Evol. **30**, 327-333. (10.1016/j.tree.2015.04.002) 25944666

[154] McNamara JM, Dall SRX, Hammerstein P, Leimar O. 2016 Detection vs. selection: integration of genetic, epigenetic and environmental cues in fluctuating environments. Ecol. Lett. **19**, 1267-1276. (10.1111/ele.12663) 27600658

[155] Trimmer PC, Houston AI, Marshall JAR, Mendl MT, Paul ES, McNamara JM. 2011 Decision-making under uncertainty: biases and Bayesians. Anim. Cogn. **12**, 465-476. (10.1007/s10071-011-0387-4) 21360119

[156] Polverino G, Palmas BM, Evans JP, Gasparini C. 2019 Individual plasticity in alternative reproductive tactics declines with social experience in male guppies. Anim. Behav. **148**, 113-121. (10.1016/j.anbehav.2018.12.014)

[157] Polverino G, Cigliano C, Nakayama S, Mehner T. 2016 Emergence and development of personality over the ontogeny of fish in absence of environmental stress factors. Behav. Ecol. Sociobiol. **70**, 2027-2037. (10.1007/s00265-016-2206-z)

[158] Stamps JA, Biro PA, Mitchell DJ, Saltz JB. 2018 Bayesian updating during development predicts genotypic differences in plasticity. Evolution **72**, 2167-2180. (10.1111/evo.13585) 30133698

[159] Stamps JA, Krishnan VV. 2014 Combining information from ancestors and personal experiences to predict individual differences in developmental trajectories. Am. Nat. **184**, 647-657. (10.1086/678116) 25325748

[160] Fischer B, van Doorn GS, Dieckmann U, Taborsky B. 2014 The evolution of age-dependent plasticity. Am. Nat. **183**, 108-125. (10.1086/674008) 24334740

[161] Stamps JA, Krishnan VV. 2017 Age-dependent changes in behavioural plasticity: insights from Bayesian models of development. Anim. Behav. **126**, 53-67. (10.1016/j.anbehav.2017.01.013)

[162] Sherratt TN, Morand-Ferron J. 2018 The adaptive significance of age-dependent changes in the tendency of individuals to explore. Anim. Behav. **138**, 59-67. (10.1016/j.anbehav.2018.01.025)

[163] Farley SS, Dawson A, Goring SJ, Williams JW. 2018 Situating ecology as a big-data science: current advances, challenges, and solutions. BioScience **68**, 563-576. (10.1093/biosci/biy068)

[164] Leonelli S. 2019 The challenges of big data biology. eLife **8**, e47381. (10.7554/eLife.47381) 30950793 PMC6450665

[165] Hauber ME, Louder MI, Griffith SC. 2021 Neurogenomic insights into the behavioral and vocal development of the zebra finch. eLife **10**, e61849. (10.7554/eLife.61849) 34106827 PMC8238503

[166] Griffin DR. 1946 Supersonic cries of bats. Nature **158**, 46-48. (10.1038/158046a0) 20991741

[167] Schevill WE, McBride AF. 1956 Evidence for echolocation by cetaceans. Deep Sea Res. **3**, 153-154. (10.1016/0146-6313(56)90096-X)

[168] Thorpe WH. 1958 The learning of song patterns by birds, with especial reference to the song of the chaffinch *Fringilla coelebs*. Ibis **100**, 535-570. (10.1111/j.1474-919X.1958.tb07960.x)

[169] Versluis M. 2000 How snapping shrimp snap: through cavitating bubbles. Science **289**, 2114-2117. (10.1126/science.289.5487.2114) 11000111

[170] Patek SN, Korff WL, Caldwell RL. 2004 Deadly strike mechanism of a mantis shrimp. Nature **428**, 819-820. (10.1038/428819a) 15103366

[171] Tinbergen N. 1963 On aims and methods of ethology. Z. Tierpsychol. **20**, 410-433. (10.1111/j.1439-0310.1963.tb01161.x)

[172] Dewsbury DA. 1989 A brief history of the study of animal behavior in North America. In Perspectives in ethology (eds P Bateson, PH Klopfer), pp. 85-122. New York, NY: Plenum Press.

